# Culture and Differentiation of Human Hair Follicle Dermal Papilla Cells in a Soft 3D Self-Assembling Peptide Scaffold

**DOI:** 10.3390/biom10050684

**Published:** 2020-04-28

**Authors:** Nausika Betriu, Claire Jarrosson-Moral, Carlos E. Semino

**Affiliations:** Tissue Engineering Research Laboratory, Department of Bioengineering, IQS-School of Engineering, Ramon Llull University, 08017 Barcelona, Spain; clairejarrossonm@iqs.url.edu (C.J.-M.); carlos.semino@iqs.url.edu (C.E.S.)

**Keywords:** self-assembling peptides, hair follicle dermal papilla cells, osteogenesis, adipogenesis, tissue engineering

## Abstract

Hair follicle dermal papilla cells (HFDPC) are a specialized cell population located in the bulge of the hair follicle with unique characteristics such as aggregative behavior and the ability to induce new hair follicle formation. However, when expanded in conventional 2D monolayer culture, their hair inductive potency is rapidly lost. Different 3D culture techniques, including cell spheroid formation, have been described to restore, at least partially, their original phenotype, and therefore, their hair inductive ability once transplanted into a recipient skin. Moreover, hair follicle dermal papilla cells have been shown to differentiate into all mesenchymal lineages, but their differentiation potential has only been tested in 2D cultures. In the present work, we have cultured HFDPC in the 3D self-assembling peptide scaffold RAD16-I to test two different tissue engineering scenarios: restoration of HFDPC original phenotype after cell expansion and osteogenic and adipogenic differentiation. Experimental results showed that the 3D environment provided by RAD16-I allowed the restoration of HFDPC signature markers such as alkaline phosphatase, versican and corin. Moreover, RAD16-I supported, in the presence of chemical inductors, three-dimensional osteogenic and adipogenic differentiation. Altogether, this study suggests a potential 3D culture platform based on RAD16-I suitable for the culture, original phenotype recovery and differentiation of HFDPC.

## 1. Introduction

Hair loss is a common problem for men and women that influences quality of life since it can result in a loss of self-esteem and depression. There is therefore considerable interest in treatments that can reverse it. The most common treatment is hair transplantation from nonbalding areas, but it is limited by the number of available donor follicles. Follicular cell implantation (FCI) is a promising cell therapy in which a patient’s own hair follicle dermal papilla cells (HFDPC) are isolated from the bulge of the hair follicle and expanded in culture to be implanted back into the scalp [1]. This therapy is based on the trichogenic properties of HFDPC, that is their ability to induce the formation of new hair follicles [2]. However, when expanded in conventional 2D monolayer culture, HFDPC lose some intrinsic properties, especially their hair inductive potency. Loss of the expression of the extracellular matrix protein versican as well as the enzyme alkaline phosphatase have been correlated with the loss of hair inductive capabilities [3]. Another hallmark of cultured HFDPC is their strong switch to α-Smooth muscle actin (αSMA) expression, which is not present in the in vivo dermal papilla [4]. Different approaches to prevent or reverse the loss of inductive potential have been described. First efforts were focused on optimizing cell cultivation medium, including the use of keratinocyte conditioned medium [5] as well as factors such as fibroblast growth factor (FGF) [6] and bone morphogenetic proteins (BMPs) [6,7]. The aggregative behavior of HFDPC led to the development of strategies involving three-dimensional cultures to reverse loss of inductive capacity. Spheroid 3D cultures using the hanging drop method or low cell-binding plates have been reported to restore, at least partially, their original phenotype, and therefore, their hair inductive ability once transplanted into a recipient skin [8,9,10]. However, spheroid cultures have some limitations, such as the inability for long-term culture, the difficulty to handle them, as well as scaling up the production. Another described strategy to restore HFDPC phenotype has been to culture them in three-dimensional scaffolds based on natural biomaterials such as cross-linked gelatin/hyaluronic acid [11] Matrigel [12] and alginate spheres [13] which could mimic their natural extracellular matrix.

Hair follicle dermal papilla cells have been mainly studied in the context of hair growth induction. However, their therapeutic potential extends far beyond inducing new hair follicles, since they have been reported to be able to differentiate to all mesenchymal lineages in 2D monolayer cell cultures [14,15,16,17,18]. Therefore, HFDPC appear to be an accessible source of stem cells that could be easily used for tissue engineering applications, since they are easy to isolate, responsive to environmental cues and may have some immune-privileged properties [19]. However, it remains to be seen how successful tissue engineering applications using HFDPC as stem cells can be since, so far, their ability to differentiate in biocompatible three-dimensional scaffolds has not been studied.

Cellular spheroids have been largely used since the 1970s to study the mechanisms of radiotherapy, chemotherapy and drug resistance in cancer models. Moreover, cellular spheroids were the first step to bring HFDPC biology to the third dimension [8,9]. However, spheroid cell culture systems present a very important limitation, that is, a poor interaction with the extracellular environment. Considering that the extracellular matrix (ECM) has a critical role in regulating biological processes such as cell proliferation, migration and differentiation, ECM analogs were introduced in 3D cell culture systems. In these systems, cells are embedded in a natural or synthetic scaffold that mimics the ECM, thus providing the chemical, mechanical, and physical cues that cells need to form physiological tissue structures in vitro. Natural scaffolds are mainly hydrogels made of natural materials or proteins, such as Matrigel^TM^ and collagen type I, which provide chemical cues, principally ECM binding motifs. However, natural scaffolds present biodegradability in vitro and therefore the maintenance of biomechanical properties along culture time is compromised. Moreover, due to its natural origin, they frequently contain residual growth factors, undefined constituents or non-quantified impurities, fact that compromises assay reproducibility [20]. In contrast, synthetic scaffolds have minimal variation from batch to batch production and provide a reproducible cellular microenvironment. Moreover, they present low biodegradability in vitro, which permits to maintain its structural and mechanical properties. In particular, synthetic peptide nanofibers-based scaffolds are characterized by 10 nm fibers and 5–200 nm pores in diameter (1,000 times smaller than cells). These dimensions allow cells to experience a truly 3D environment. Peptide nanofibers contain biologically inspired sequences, with the most relevant examples being peptide amphiphiles [21], β-hairpin peptides [22], and self-assembling peptides [23]. Inspired by tandem repeats found in natural proteins, such as the sequence EAK16 in the yeast protein Zuotin [24] or Lysβ-21 in the egg white Lysozyme [25], a new generation of self-assembling peptides such as DN1 [25] RAD16-I [23] and KLD12 [26] were rationally designed.

In the present work, we have used the self-assembling peptide scaffold (SAPS) RAD16-I to culture and differentiate human hair follicle dermal papilla cells. RAD16-I is a synthetic peptide constituted by the sequence AcN-(RADA)_4_-CONH_2_, that alternates hydrophilic and hydrophobic amino acids [23]. The peptide undergoes self-assembly into a nanofiber network with antiparallel β-sheet configuration by increasing ionic strength or adjusting pH to neutrality. Importantly, the nanoscale architecture of the fiber network (around 10 nm diameter and 50–200 nm pore size) allows cells to experiment a truly 3D environment. Non-covalent interactions permit cell growth, migration, contact with other cells, shape changes, and a proper exposition of membrane receptors [23,27,28]. Moreover, stiffness can be controlled by changing peptide concentration, which enables their use in different tissue engineering applications. Unlike natural scaffolds, the constituents of this synthetic peptide are well defined and do not present variations from batch to batch. This synthetic hydrogel provides a non-instructive environment, but it can be easily modified to incorporate signaling motifs or functional biomolecules [29,30]. Moreover, RAD16-I has been shown to be biodegradable and non-toxic in vivo [31]. Several studies have reported the capacity of RAD16-I to support growth and maintenance of multiple cell types such as endothelial cells [29], hepatocytes [32,33], embryonic [31], and somatic stem cells [34], as well as osteogenic [35,36] and chondrogenic [37,38,39] differentiation of different cell types. 

The goal of the present study is to culture and restore human hair follicle dermal papilla cells phenotype in RAD16-I scaffold, as well as to differentiate them toward osteogenic and adipogenic lineages to test its multipotent capacity in a three-dimensional environment. Experimental results show that HFDPC cultured in the soft RAD16-I hydrogel were able to elongate and stablish cell-cell interactions while contracting the matrix. Moreover, expression of HFDPC signature markers such as alkaline phosphatase, versican and corin was increased after seven days of culture within the peptide hydrogel. Finally, RAD16-I has been shown to support three-dimensional osteogenic and adipogenic differentiation of dermal papilla cells.

## 2. Materials and Methods 

### 2.1. 2D Culture 

Hair Follicle Dermal Papilla Cells (HFDPC) (C12071, Promocell, Heidelberg, Germany) were cultured at 10,000 cells/cm^2^ from passage 2 to passage 6 in 75 cm^2^ T-flasks in Follicle Dermal Papilla Cell Growth Medium (C26501, Promocell), which contains fetal calf serum, bovine pituitary extract, bFGF and insulin, supplemented with L-Glutamine (X0550, Biowest, Nuaillé, France) and 1% (*w*/*v*) Penicillin/Streptomycin (P/S) (L0022, Biowest). Human Foreskin Fibroblasts (HFF) and HeLa cells were cultured at 10,000 cells/cm^2^ for not more than 10 passages in DMEM (DMEM-HXA, Capricorn Scientific, Ebsdorfergrund, Germany) supplemented with 10% FBS (S1810; Biowest), L-glutamine and P/S. Adipose-derived stem cells (ADSC) were cultured from passage 2 to passage 6 at 10,000 cells/cm^2^ in ADSC Basal Medium (PT-3273, Lonza, Basel, Switzerland) with SingleQuots^TM^ Growth Supplement Kit (PT-4503, Lonza). Cultures were maintained at 37 °C and 5% CO_2_ in a humidified atmosphere and passaged at 80% confluency. 

### 2.2. 3D Culture Technique in the Self-Assembling Peptide Scaffold RAD16-I

The protocol for cell encapsulation into self-assembling peptides scaffolds was previously described in detail [40]. Briefly, the peptide RAD16-I (commercially available at 1% in water, PuraMatrix^TM^, 354250, Corning, New York, NY, USA) was diluted to a final concentration of 0.3% (*v*/*v*) in 10% (*w*/*v*) sucrose (S0389, Sigma-Aldrich St. Louis, MO, USA) and sonicated during 30 min. Meanwhile, cells were harvested by trypsinization and resuspended to 4 × 10^6^ cells/mL in 10% (*w*/*v*) sucrose, which is an isotonic and non-ionic medium that allows cell maintenance and avoids peptide assembly during the encapsulation process Then, the cell suspension was mixed with an equal volume of RAD16-I peptide solution to obtain a mixture of 2 × 10^6^ cells/mL and 0.15% RAD16-I. Next, 40 µL of cell-peptide suspension (80,000 cells) were loaded into wells of a 48-well plate previously equilibrated with 500 µL of culture medium, which induced the peptide spontaneous self-assembly. The peptide was let gel in the flow cabinet for 20 min and the plate was placed in the incubator for 1 h. Medium was changed twice to favor the leaching of sucrose. 3D cultures were maintained in DMEM supplemented with 10% FBS, L-glutamine, and P/S at 37 °C and 5% CO_2_ in a humidified atmosphere. Medium was changed every other day.

### 2.3. Construct Morphology Evaluation

The macroscopic morphology of HFDPC 3D constructs was monitored with a stereoscopic microscope (Nikon SMZ800, Nikon, Tokyo, Japan) and the area was measured with ImageJ software. The contraction degree was calculated as the percentage area decrease respect to the initial one.

### 2.4. Cell Viability Assessment

#### 2.4.1. Live/Dead Staining

Cell viability in 3D constructs was qualitatively determined using the Live/Dead Viability/Cytotoxicity kit for mammalian cells (L3224, Invitrogen, Waltham, MA, USA). Constructs were rinsed with warm 1X PBS and incubated during 15 min (37 °C and 5% CO_2_ in a humidified atmosphere) with a fresh solution of 2 µM calcein-AM and 2 µM ethidium homodimer-1 in PBS. Samples were then washed three times with 1X PBS and visualized under the fluorescence microscope (Nikon Eclipse TE2000-1 epifluorescent microscope).

#### 2.4.2. MTT Assay

MTT [3-(4,5-dimethylthiazol-2-yl)-2,5-diphenyltetrazolium bromide] assay (M5655, Sigma-Aldrich) was used to assess cell viability in 3D constructs. Cell culture medium was aspirated and 500 μL of MTT reagent was added to a final concentration of 0.5 mg/mL in culture medium. Samples were incubated during 3 h (37 °C and 5% CO_2_ in a humidified atmosphere). Subsequently, MTT solution was removed and the constructs were lysed with 200 µL of DMSO (D8418, Sigma-Aldrich). Absorbance was read at 590 nm using a microplate reader (Biotek Epoch^TM^, Biotek, Winooski, VT, USA).

### 2.5. Osteogenic and Adipogenic Differentiation

HFDPC 3D cultures were induced 24 h after cell encapsulation. Cultures were maintained under adipogenic and osteogenic medium for 21 days. Control medium consisted of DMEM supplemented with 10% FBS, L-Glutamine and P/S. Osteogenic medium consisted on control medium supplemented with 50 µM L-ascorbic acid 2-phosphate (A8960, Sigma-Aldrich), 100 nM Dexamethasone (D8893, Sigma-Aldrich) and 10 mM β-Glycerol-2-phosphate (G9422, Sigma-Aldrich) [17]. Adipogenic medium consisted on control medium supplemented with 10 µg/mL insulin (I9278, Sigma), 100 nM Dexamethasone (D8893, Sigma-Aldrich), 0.2 mM Indomethacin (I7378, Sigma-Aldrich) and 0.5 mM isobutyl-1-methylxanthin (I7018, Sigma-Aldrich) [17]. 

### 2.6. Staining for Phenotype Assessment

#### 2.6.1. Alkaline Phosphatase Staining

Briefly, cultures were rinsed with 1X PBS and fixed with 4% formaldehyde during 15 min. Cells were washed with PBS and permeabilized with 0.1% Triton X-100 during 30 min. Afterwards, cultures were extensively washed with a buffer containing no phosphate (0.1 M Tris Base, 100 mM NaCl, 5 mM MgCl_2_, pH 9.5) and incubated during 10 min with the substrate solution containing nitrotetrazolium blue (NBT) and 5-bromo-4-chloro-3-indolyl phosphate (BCIP) (NBT/BCIP Stock Solution, 11681451001, Sigma-Aldrich). The reaction was stopped by washing the samples with dH_2_O. The formation of a purple precipitate indicated alkaline phosphatase activity.

#### 2.6.2. Toluidine Blue Staining

For the detection of glucosaminoglycans (GAGs) 2D and 3D cultures were washed with 1X PBS and fixed with 4% formaldehyde during 15 min. Cells were then washed with 1X PBS and incubated with 0.05% (*w*/*v*) toluidine blue (89640, Sigma-Aldrich) in dH_2_O during 20 min. Finally, samples were washed several times overnight with dH_2_O and visualized with a stereoscopic microscope (Nikon SMZ800).

#### 2.6.3. Immunocytochemistry

Cells in 2D cultures and 3D constructs were fixed with 4% formaldehyde during 15 min and washed 3 times with 1X PBS. Next, samples were incubated with 3% H_2_O_2_ in PBS during 30 min to block endogenous peroxidases. Cultures were blocked with 5% BSA/0.1% Triton X-100 in PBS during 2 h and incubated overnight at 4 °C with primary antibody mouse anti-αSMA (sc-32251, scbt, Dallas, TX, USA) at 1/50. Afterwards, cells were washed with blocking buffer and incubated 2 h with Goat anti mouse-HRP secondary antibody (ab205719, abcam, Cambridge, United Kingdom) at 1/2000. Finally, samples were incubated with DAB substrate (8801-4965-72, Invitrogen) during 10 min. The specific bound of DAB to its enzyme HRP generated a brown precipitate that could be visually observed under a stereoscopic microscope (Nikon SMZ800).

#### 2.6.4. Immunofluorescence

Cells in 2D cultures and 3D constructs were fixed with 4% formaldehyde for 15 min and washed 3 times with 1X PBS. Cultures were blocked with 5% BSA/0.1% Triton X-100 in PBS during 2 h and incubated overnight at 4 °C with primary antibodies. Next, cells were washed with blocking buffer and incubated for 2 h with secondary antibodies. Finally, cells were counterstained with Phalloidin-TRITC and DAPI for cytoskeleton and nuclei visualization using fluorescence microscopy (Nikon Eclipse TE2000-1 epifluorescent microscope and Leica Thunder Imager widefield microscope). Primary antibodies and dilutions used were mouse anti-versican (MA5-27638, Invitrogen) at 1/100, rabbit anti-OPN (ab63856, abcam) at 5 μg/mL and rabbit anti-FABP4 (ab23693, abcam) at 4 μg/mL. Secondary antibodies used were Donkey anti mouse-A488 (ab150105, abcam) and Goat anti rabbit-A647 (ab150079, abcam) at 1/500.

#### 2.6.5. Von Kossa Staining 

HFDPC cultured for 21 days under control and osteogenic medium were fixed with 4% formaldehyde during 15 min and washed with 1x PBS. Then, samples were extensively washed overnight with dH_2_O to completely remove phosphates. Afterwards, samples were incubated with a 5% (*w*/*v*) silver nitrate (209139, Sigma-Aldrich) solution for 1 h in the dark. Finally, 3D constructs were washed with dH_2_O and left under bright light during 10 min. Samples were visualized under a stereoscopic microscope (Nikon SMZ800) to detect the presence of calcium mineralization. 

#### 2.6.6. Nile Red Staining 

HFDPC cultured for 21 days under control and adipogenic medium were fixed with 4% formaldehyde during 15 min and washed with 1X PBS. Then constructs were washed with dH_2_O followed by 30 min incubation with 35% glycerol. Finally, samples were stained with 2.5 μg/mL Nile red (19123, Sigma-Aldrich) in 75% glycerol for 10 min. Afterwards, cells were washed with dH_2_O before being counterstained with DAPI.

### 2.7. RNA Extraction, cDNA Synthesis and PCR

RNA was extracted from the samples using Qiagen RNeasy Plus Mini Kit (74134, Qiagen, Hilden, Germany) and genomic DNA was removed using RNase-Free DNase Set (79254, Qiagen). RNA purity and concentration were quantified using Epoch Take3 microvolume plate (Biotek) and cDNA was synthesized using TranscriptME RNA kit (RT31, Blirt, Gdansk, Poland). PCR was carried out with 25 ng of cDNA templates using 2X PCR TaqNova-RED (RP85T, Blirt), which included all the components needed to perform cDNA amplification. The primers used are listed in Table 1. The PCR (Veriti^TM^ Thermal Cycler, Applied Biosystems, Foster City, CA, USA) was carried out under the following conditions: 5 min at 94 °C followed by 30 cycles of 15 s at 94 °C, 30s at 55 °C, and 15 s at 72 °C, and a final extension step of 5 min at 72 °C. Finally, DNA electrophoresis in 2% agarose gels was performed with PCR products to determine the relative expression of the selected genes.

### 2.8. Western Blot

Briefly, 3D constructs were lysed with RIPA buffer (R0278; Sigma-Aldrich) containing protease inhibitor cocktail (11836153001, Roche, Basel, Switzerland). Total protein was quantified with BCA protein assay kit (39228, Serva, Heidelberg, Germany) and 5 μg of total protein were loaded into a polyacrylamide gel and run by applying 225 V during 40 min. Afterwards, proteins were transferred to a PVDF membrane by applying 40 V during 2 h at room temperature. The membrane was then blocked with 4% (*w*/*v*) nonfat powdered milk in 0.2% PBS-Tween for 1 h and then incubated with primary antibodies rabbit anti-FABP4 (ab23693, abcam) at 2 μg/mL and rabbit anti-vinculin (700062, Invitrogen) at 1/1000 during 1 h at RT. Finally, the membrane was incubated with Goat anti rabbit-HRP (ab6721, abcam) at 1/1000 for 1 h at RT and revealed for HRP detection with SuperSignal West Pico Chemiluminescent Substrate (34080; Thermo Fisher Scientific, Waltham, MA, USA). Chemiluminescent images were taken in the ImageQuant^TM^ LAS 4000 mini (GE HealthCare, Chicago, IL, USA). 

## 3. Results

### 3.1. HFDPC Culture in the 3D Self-Assembling Peptide Scaffold RAD16-I

**Figure 1:**
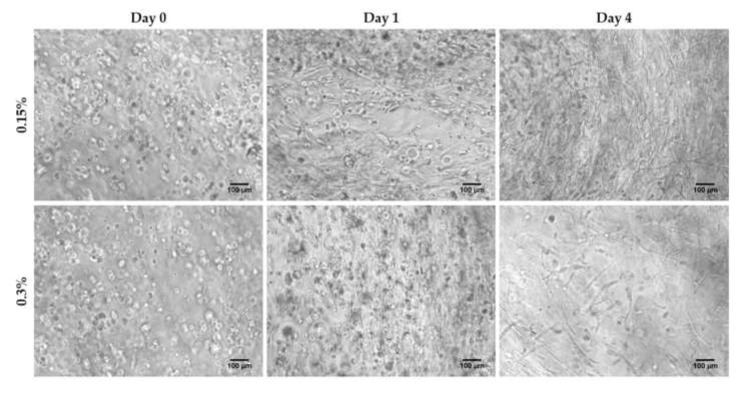
HFDPC cultured in 3D self-assembling peptide scaffold RAD16-I at 0.15% and 0.3% peptide concentration. Scale bar 100 µm.

Human hair follicle papilla cells were expanded to passage 6 in conventional 2D monolayer culture and encapsulated in 3D scaffolds at two different RAD16-I peptide concentrations: 0.15%, which corresponds to a stiffness of approximately 120 Pa, and 0.3%, corresponding to approximately 510 Pa, as determined by Sieminski et al. [41] by rheological analysis. Just after encapsulation (day 0), cells presented a round morphology at both peptide concentrations. At day 1, some cells started to elongate cellular processes in soft hydrogels, while remained round in stiffer scaffolds. After four days in culture, cells within soft scaffolds presented an extended shape and interacted between them, creating a highly interconnected cellular network. On the contrary, in stiffer gels, cellular network at day 4 was less dense, with some cells remaining, yet with a round shape (Figure 1).

**Figure 2:**
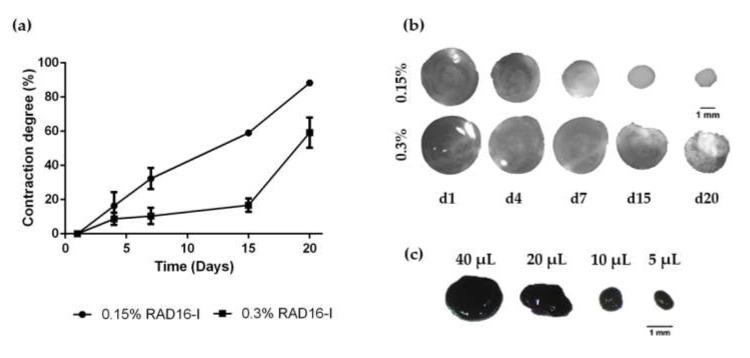
Matrix contraction of HFDPC in RAD16-I scaffolds. (**a**) Matrix contraction degree in soft (0.15%) and stiff (0.3%) hydrogels; (**b**) Macroscopic view of 0.15% and 0.3% peptide concentration 3D constructs along culture time; (**c**) 0.15% RAD16-I constructs at day 7 of culture stained by MTT. Construct size can be modulated by changing the initial seeding volume.

The extended cell morphology, the presence of cell-cell interactions at each time point and the stiffness of the scaffold correlated with the decrease in the macroscopic size of the 3D constructs (Figure 2a,b). In soft scaffolds at 0.15% peptide concentration, matrix contraction by the cells was faster and more pronounced, presenting a contraction degree of approximately 30% in seven days, and it continued contracting along culture time (approximately 90% of contraction after 20 days in culture). Moreover, soft 3D constructs were fragile and easy to break during the first days of culture, but the contraction process led to more packed and stiffer structures, which were easy to manipulate with tweezers without breaking. On the other hand, in stiffer matrices at 0.3% peptide concentration macroscopic changes were not noticeable until day 15 of culture (approximately 20% of contraction) (Figure 2a,b).

Contraction is one of the mechanisms used by cells to remodel their surroundings. This is a natural process when mesenchymal-type cells interact with the matrix fibers and contract their actomyosin cytoskeleton, producing an organized and macroscopic effect that is visualized as a contraction of the 3D construct. Cellular contraction in non-instructive synthetic matrices is usually due to the interaction between integrins and ECM proteins that decorate the scaffold, either from exogenous origin (for example from the fibronectin present in FBS used to supplement cell culture medium) or endogenous origin (synthesized and secreted by the cells). This matrix contraction is progressive and depends on culture conditions, the cell density and the initial matrix stiffness. 

One advantage of using this biomaterial for cell encapsulation, is that the initial 3D construct volume and therefore, the final size of the constructs after matrix contraction can be easily modulated. In this work, we have used 40 µL-gels with a cell density of 2 × 10^6^ cells/mL (80,000 cells/construct)—which is enough to allow cell–cell interactions—because they are easier to handle than smaller gels. However, smaller constructs (for example 20 µL-gels; 40,000 cells, 10 µL-gels; 20,000 cells or 5 µL-gels; 10,000 cells) can also be obtained by simply changing their initial volume (Figure 2c). Hence, the self-assembling peptide scaffold RAD16-I offers a platform which makes it possible to scale the size of 3D cellular constructs.

**Figure 3:**
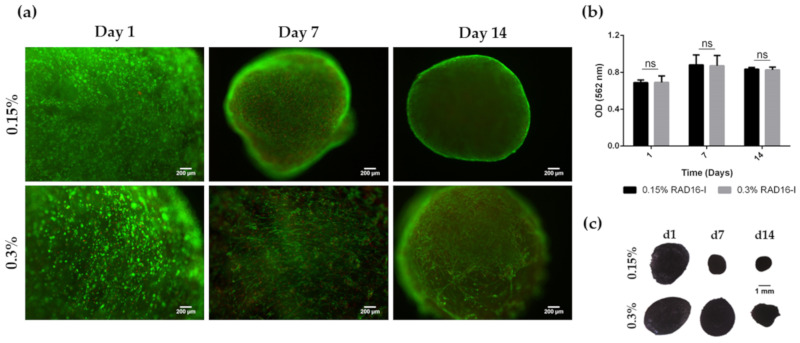
Viability of HFDPC cultured in the 3D self-assembling peptide scaffold RAD16-I at 0.15% and 0.3% peptide concentration. (**a**) Fluorescent images of Live/Dead staining. Scale bar: 200 µm; (**b**) MTT absorbance values of 3D HFDPC constructs at different time points in culture; (**c**) Macroscopic view of 3D constructs after MTT incubation.

Finally, the viability of HFDPC within 3D scaffolds at both peptide concentrations was evaluated with the qualitative Live/Dead staining and the quantitative MTT assay (Figure 3). Fluorescent images show that most of the cells were alive after 14 days of culture (Figure 3a). This result was confirmed by MTT assay, with constant absorbance values along culture time (Figure 3b) and strong purple staining of 3D constructs after MTT incubation (Figure 3c). Because no differences regarding cell viability were found between both peptide concentrations and because soft hydrogels allowed the formation of a richer cellular network than the stiffer ones, we decided to use this peptide concentration for further experiments.

### 3.2. Analysis of HFDPC Signature Markers

**Figure 4:**
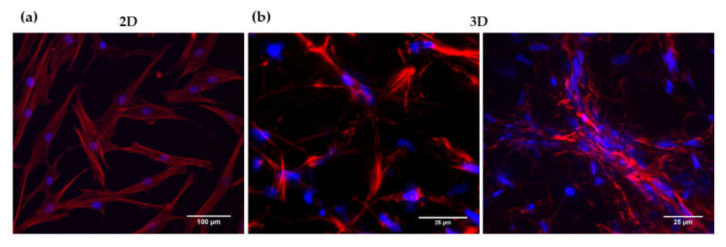
DAPI/Phalloidin staining of HFDPC in (**a**) 2D and (**b**) 3D cultures.

HFDPC cultured in 2D presented a flat fibroblast-like shape with the presence of actin stress fibers (Figure 4a). On the other hand, HFDPC in the 3D milieu spread and adopted a lengthened shape forming an interconnected cellular network. In some areas, cell aggregation in clusters could be observed (Figure 4b). This cellular elongation and network formation were possible due to the permissive microenvironment provided by RAD16-I hydrogel, which has non-covalent interactions between its nanofibers.

**Figure 5:**
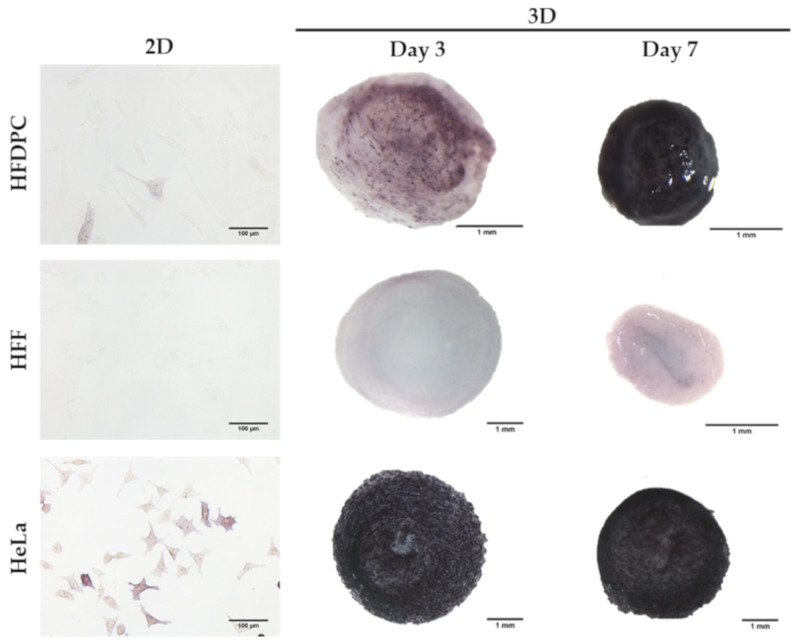
Alkaline phosphatase staining in 2D and 3D cultures. HFDPC were negatively stained for ALP activity in 2D cultures. The expression of ALP was recovered once encapsulated in RAD16-I 3D scaffolds. Human Foreskin Fibroblasts (HFF) were used as a negative control and HeLa cells as a positive control for ALP staining.

We analyzed alkaline phosphatase (ALP) activity in 2D and 3D cultures, the expression of which has been positively correlated with the hair inductive potential of HFDPC [3]. Human foreskin fibroblasts (HFF) were used as a negative control, since they are isolated from a non-bearing hair skin, while HeLa cells were used as a positive control. In 2D cultures, HFDPC were barely stained for ALP activity, which agrees with previous work reporting the decrease of ALP expression during 2D culture time [3,8]. Once encapsulated within RAD16-I 3D scaffolds, HFDPC recovered a certain degree of ALP activity by day 3 of culture, while constructs at day 7 presented a strong purple color, positively associated with ALP activity (Figure 5). 

**Figure 6:**
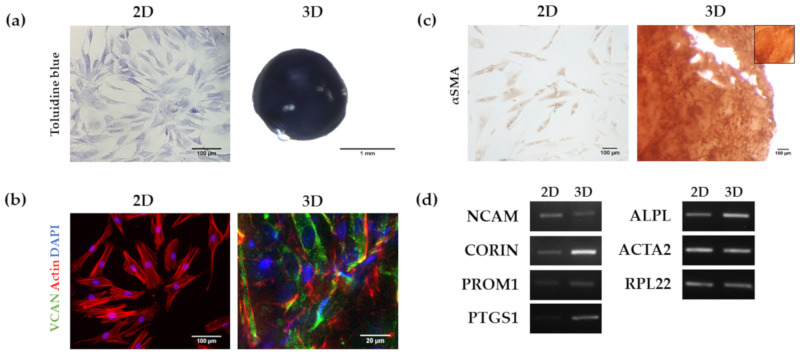
Analysis of HFDPC markers in 2D and RAD16-I 3D cultures. (**a**) Toluidine blue staining for the detection of glycosaminoglycans; (**b**) Versican (VCAN) immunofluorescence. VCAN was absent in 2D cultures but its expression was recovered in 3D cultures as shown by immunofluorescence detection; (**c**) α Smooth Muscle Actin (αSMA) immunocytochemistry. αSMA was detected in both 2D and 3D cultures. The inner box represents a negative control (incubation with secondary antibody only); (**d**) Agarose gel of Reverse-transcription PCR products including Neural Cell Adhesion Molecule (*NCAM*), Corin (*CORIN*), Prominin-1 (*PROM1*), Prostaglandin endoperoxidase synthase 1 (*PTGS1*), alkaline phosphatase (*ALPL*) and α-Smooth Muscle Actin (*ACTA2*).

Proteoglycans (PGs) expression such as versican and perlecan together with a variety of GAGs like heparan sulphate (HS), chondroitin sulphate (CS) and keratan sulphate (KS) has been described in the in vivo dermal papilla of the hair follicle. Moreover, loss of versican expression during 2D expansion has been correlated with a loss of inductive capacity [42,43]. We stained HFDPC 2D and RAD16-I 3D cultures with toluidine blue for GAGs detection (Figure 6a) and found positive staining in both cultures, indicating the presence of sulphated GAGs. Immunodetection of the proteoglycan versican (VCAN) showed that, although absent from 2D cultures, its expression was recovered after seven days in 3D culture in RAD16-I scaffold (Figure 6b). We also analyzed αSmooth muscle actin (αSMA) expression, which is a marker of HFDPC cultured in vitro but absent in the in vivo dermal papilla [4]. In concordance with previous reports, HFDPC in 2D cultures were positively stained by αSMA, but failed to downregulate its expression after seven days of culture in the 3D peptide hydrogel (Figure 6c). 

We finally analyzed the expression of different HFDPC markers at the transcriptional level by Rt-PCR (Figure 6d). RNA was extracted from passage 6 cells growing in 2D or maintained for 7 days in 3D cultures and retrotranscribed into cDNA, which was afterwards amplified by PCR. We found that the expression of Corin (*CORIN*) [44], Prominin-1 (*PROM1*) [45], Prostaglandin endoperoxidase synthase 1 (*PTGS1*) [46], and alkaline phosphatase (*ALPL*) in RAD16-I 3D cultures was upregulated compared to 2D cultures. On the other hand, NCAM [47] was decreased in 3D cultures compared to 2D. Lastly, and consistent with the immunostaining, α-smooth muscle actin gene (*ACTA2*) [4] expression remained constant in both 2D and 3D cultures. 

### 3.3. Osteogenic and Adipogenic Differentiation

**Figure 7:**
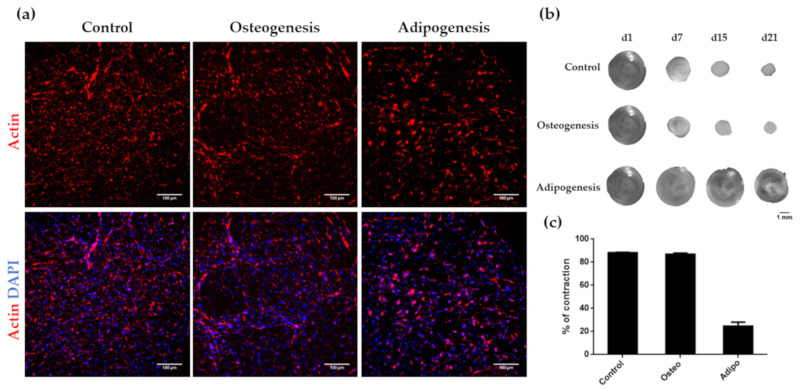
Influence of osteogenic and adipogenic induction on cell morphology and matrix contraction in HFDPC. (**a**) DAPI/Phalloidin staining of 3D cultures under control, osteogenic and adipogenic medium; (**b**) Construct contraction along culture time; (**c**) Contraction degree after 21 days of culture under control, osteogenic and adipogenic medium.

During years, hair follicle dermal papilla cells have been mainly studied in the context of hair growth induction and in the development of skin equivalents [48,49,50,51,52]. However, their therapeutic potential extends far beyond hair and skin tissue engineering applications, since these cells have been reported to differentiate to various mesenchymal lineages in 2D monolayer cell cultures [14,15,16,17,18]. However, no differentiation studies of HFDPC have been performed in 3D scaffold-based cultures. It is well known that the 3D environment mimics better the in vivo conditions and the natural tissue, and therefore, the use of 3D scaffolds should be considered when assessing the differentiation potential of different cell types. In the present work, we analyzed osteogenic and adipogenic differentiation potential of HFDPC in the 3D non-instructive environment provided by RAD16-I hydrogel. Similar to the control (Figure 7a, left), cells under osteogenic induction formed a highly interconnected network with elongated cells (Figure 7a, middle), that contracted the matrix up to a 90% by day 21 of culture (Figure 7b,c). On the other hand, cells under adipogenic medium had a more rounded shape, with fewer cellular interconnections (Figure 7a, right). In this case, the cells barely contracted the peptide matrix (Figure 7b,c).

**Figure 8:**
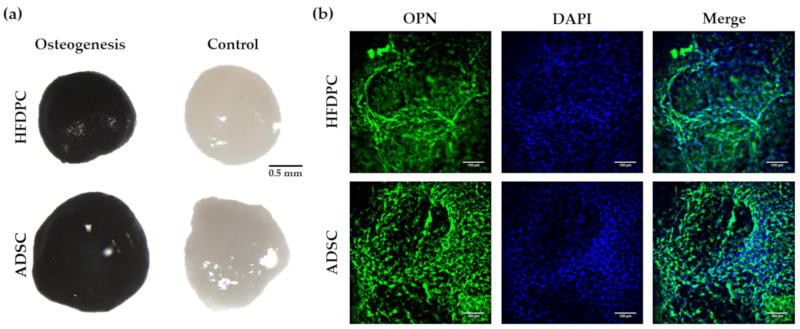
Characterization of osteogenic phenotype of HFDPC and ADSC cultured in RAD16-I scaffold for 21 days. (**a**) von Kossa staining (calcium mineralization) of 3D constructs cultured in control and osteogenic medium; (**b**) OPN immunofluorescence of 3D constructs under osteogenic induction.

We next analyzed different osteogenic and adipogenic markers in 3D induced HFDPC, and we compared them to adipose-derived stem cells (ADSC) which have been widely used in tissue engineering applications, and have already been proved to differentiate into these lineages in 3D environments [53,54]. We performed von Kossa staining on HFDPC and ADSC cultured under control and osteogenic medium to detect the presence of calcium mineralization, which is the process that occurs in late osteogenesis. Thereby, 3D constructs under osteogenic induction medium became highly stained in black color, due to the presence of calcium mineralization, suggesting a terminal differentiation (Figure 8a). No staining was detected in 3D constructs under control medium, meaning that the osteogenic differentiation was not a spontaneous process. We also detected the presence of osteopontin (OPN) (Figure 8b), which is an extracellular matrix protein synthesized by osteoblasts. No OPN staining was detected in control conditions (results not shown). Qualitatively, no differences between both HFDPC and ADSC cell types regarding osteogenic differentiation efficiency were detected.

**Figure 9:**
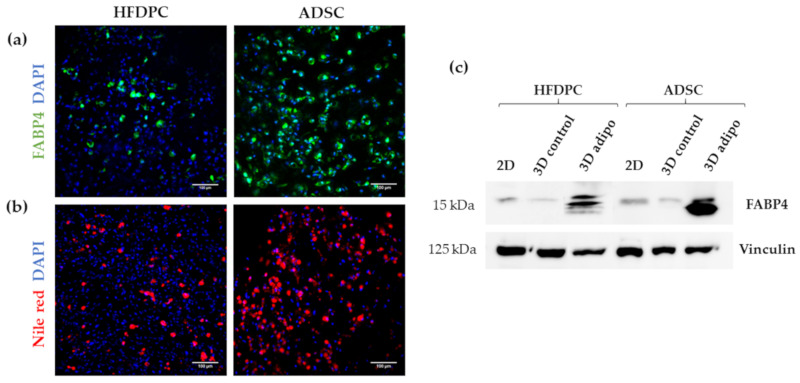
Characterization of adipogenic phenotype of HFDPC and ADSC cultured in RAD16-I scaffold for 21 days. (**a**) FABP4 immunofluorescence of 3D constructs under adipogenic induction; (**b**) Nile red staining of 3D constructs under adipogenic induction; (**c**) FABP4 western blot of HFDPC and ADSC cultured in 2D cultures and in 3D RAD16-I scaffold under control and adipogenic medium. Vinculin was used as an internal control. The image is representative of three replicates.

To test adipogenic differentiation we analyzed the expression of Fatty Acid-Binding Protein 4 (FABP4), which is a carrier protein for fatty acids that is expressed in adipocytes. We found positive FABP4 staining in both cell types under adipogenic induction (Figure 9a) and detected also the presence of intracellular lipid droplets by Nile red staining (Figure 9b). However, we observed that the presence of FABP4 and lipid droplets was much higher in ADSC compared to HFDPC, suggesting that adipogenic differentiation efficiency was greater in ADSC than in HFDPC. This result was confirmed by Western Blot analysis of FABP4 (Figure 9c), which was detected in 3D cultures of both cell types under adipogenic induction. However, a more intense band was detected for ADSC compared to HFDPC. Faint FABP4 bands could be also observed in 2D cultures and 3D control cultures in both cell types.

## 4. Discussion

During morphogenesis, mesenchymal condensation (MC) is a fundamental step for proper tissue development and function. Condensates of cells from the mesenchyme precede the formation of organs and tissues such as bones, limbs, muscles and hair follicles. In fact, the dermal papilla of the hair follicle arises from a local condensation (dermal condensate, DC) of fibroblasts [2,55], where cell migration as well as cell–cell interactions play a critical role. Thus, cell–cell interactions and communication are crucial for proper follicle morphogenesis. Because growing HFDPC in 3D spheroids allows the recovering of some inductive ability that is lost during in vitro expansion [8,9], it has been suggested that cellular condensation is closely linked with HFDPC maintenance. Thus, among current 3D cell culture methods, spheroid formation by the hanging drop method has shown to be a promising technique to restore, at least partially, HFDPC phenotype. However, cell spheroids have some drawbacks, such as poor interactions with the ECM, the impossibility of long-term culture due to the inability of medium changes, and volume limitations for each drop, which may result in insufficient nutrition. Other groups have demonstrated recovery of trichogenic nature in 3D scaffold-based cultures such as Matrigel [12], alginate beads [13], and cross-linked gelatin/hyaluronic acid [11]. The advantage of natural scaffolds is that they may provide efficient cell adhesion sites and biological signals, but usually contain undefined constituents, compromising assay reproducibility. Moreover, their biological source often prevents them from any application in humans. On the other hand, synthetic scaffolds are well defined and provide a controllable microenvironment which can be easily tuned with molecular cues. It is well known that matrix compliance plays a key role in cellular functions such as spreading, proliferation, migration, and differentiation [56]. However, in most synthetic hydrogels, cells remain entrapped, and spreading and cellular interconnections are impeded. Therefore, soft hydrogels that provide an unrestricted environment where cells can spread and create a cellular network are valuable material alternatives. In the present work, cells were embedded in a synthetic self-assembling peptide hydrogel that allowed homogenous encapsulation, cell elongation and cell-cell interactions (Figure 1). With such unrestrictive environment, high number of cellular connections were stablished, which were translated into a matrix contraction (Figure 2), that could somehow resemble the dermal condensation that occurs during dermal papilla development. Moreover, this platform was proven to support viability for prolonged culture time, regardless of the initial matrix stiffness (Figure 3). 

We also characterized different HFDPC signature markers which have been described to be relevant in hair follicle inductivity. Alkaline phosphatase activity was scarcely detected in 2D after in vitro expansion, but 3D RAD16-I constructs at day 7 of culture became strongly stained for ALP activity (Figure 5). At a transcriptional level, *ALPL* was upregulated in 3D RAD16-I cultures compared to 2D (Figure 6d). Another hallmark of the in vivo dermal papilla is the presence of high amounts of GAGs such as heparan sulphate (HS), chondroitin sulphate (CS) and keratan sulphate (KS) [43]. Accordingly, HFDPC in 3D RAD16-I cultures became intensely stained by toluidine blue, which stains for sulphated GAGs. We also analyzed versican expression, a chondroitin sulphate proteoglycan which is expressed in the dermal papilla of hair follicles in the growing phase (anagen) and is required for normal human hair growth [42,57]. In this study, we show that although absent from 2D cultures, versican expression was detected in 3D RAD16-I cultures (Figure 6b). Further, 3D culture in RAD16-I scaffold allowed the upregulation of other HFDPC signature genes that are diminished in 2D culture, such as *Corin*, *Ptgs-1* and *Prominin-1* (Figure 6d). *NCAM* expression was found to be downregulated in 3D cultures compared to 2D, which is in concordance with previous reports using spheroid cell cultures [8]. Regarding αSMA, which is strongly expressed in vitro but absent in the in vivo dermal papilla [4], this 3D platform failed to promote its downregulation at a protein (Figure 6c) and gene level (*ACTA2*) (Figure 6d). In fact, scientific reports regarding αSMA expression in cultured HFDPC are controversial since some works report the loss of αSMA in spheroid cell cultures by the hanging drop method [8] but others show that αSMA is present in 3D cell spheroids formed by hanging drop [10], inside Matrigel [12], or on top of poly(ethylene-co-vinyl alcohol) membranes [58].

Many types of adult stem cells have been reported over several decades. The ideal stem cells that would be useful in tissue engineering need to be easily obtained in large numbers, safe to implant, and able to differentiate into the cells needed. Potential adult stem cells for use in tissue engineering, such as mesenchymal stem cells, hematopoietic stem cells and skin stem cells are easy to access, and can relativity be obtained in large numbers [59]. Moreover, they are safe because they are harvested from adult tissues, in contrast to iPS cells [60] whose tumorigenic potential often prevents their clinical application. The skin is a large self-renewing organ that contains different stem cells niches, one of them, the hair follicle dermal papilla, located in the bulge of the hair follicle. During years, follicle dermal papilla cells have been studied in the context of hair growth induction, epidermal regeneration in response to skin injury, as well as in the development of skin equivalents [48,49,50,51,52]. However, it seems that HFDPC could also be used as a source of stem cells for tissue engineering applications since they have been reported to be able to differentiate to all mesenchymal lineages in 2D monolayer cell cultures [14,15,16,17,18]. First studies showed that rodent dermal papilla cells have a broad differentiation potential, similar to bone marrow derived mesenchymal stem cells [16,61]. Later, other groups demonstrated that dermal papilla cells from human hair follicles exhibited a mesenchymal stem cell immunophenotype, and differentiated to all mesenchymal lineages [14,15]. Consequently, these cells are now in the focus of interest as an accessible source of stem cells, that could be easily used for tissue engineering applications. However, even their mesenchymal lineages differentiation potential has been tested in 2D monolayer cultures, no studies have been performed in 3D scaffold-based cultures. In this work, we have for the first time demonstrated osteogenic and adipogenic differentiation of HFDPC in a 3D scaffold-based culture. In particular, von Kossa staining, which detects calcium mineralization, was highly positive in 3D constructs under osteogenic medium, while no staining was detected in control conditions (Figure 8a). Moreover, osteopontin (OPN) was strongly detected in cells under osteogenic conditions (Figure 8b), suggesting that HFDPC cultured in the 3D RAD16-I hydrogel present a high osteogenic differentiation efficiency, similar to ADSC. Regarding adipogenesis, we detected the presence of FABP4 (Figure 9a,c) as well as some lipid droplets (Figure 9b) in HFDPC 3D constructs under adipogenic medium. However, adipogenic differentiation efficiency was low compared to ADSC (Figure 9a–c).

In summary, the present study reports promising results involving the use of HFDPC in different tissue engineering scenarios, revealing the versatility of RAD16-I scaffold depending on the culture conditions provided.

## 5. Conclusions

In the present work, we have cultured hair follicle dermal papilla cells in the soft 3D self-assembling peptide scaffold RAD16-I to test two different tissue engineering scenarios: restoration of HFDPC original phenotype after cell expansion and induction of HFDPC to osteogenic and adipogenic lineage commitment. We show that the 3D nanometric environment that provides this synthetic matrix allows the upregulation of signature markers involved in hair follicle inductivity, such as alkaline phosphatase and versican, and therefore the partial restoration of HFDPC original phenotype. Moreover, it also permits, under the appropriate medium conditions, to direct osteogenic and adipogenic differentiation, as evidenced by matrix mineralization and osteopontin expression in the case of osteogenesis, and FABP4 and lipid droplets accumulation in adipogenesis.

## Figures and Tables

**Table 1:** List of amplification primers.

Gene	Forward/Reverse	Sequence (5′–3′)	Size (bp)	Amplicon (bp)
NCAM	Forward	5′-TAAGCTCGAAGGGCAGATGG-3′	20	142
	Reverse	5′-GAGCCTGATCTCTGGTTTCC-3′	20	
CORIN	Forward	5′-GGCTGTGTCCTCATTGCCAA-3′	20	146
	Reverse	5′-GTCTTCACAAAGCGTGTCTGC-3′	21	
PROM1	Forward	5′-TTCTTGACCGACTGAGACCCA-3′	21	99
	Reverse	5′-TCATGTTCTCCAACGCCTCTT-3′	21	
PTGS1	Forward	5′-GGCACCAACCTCATGTTTGC-3′	20	146
	Reverse	5′-TGACGCTCCAGATTGTCTCC-3′	20	
ALPL	Forward	5′-ATGGGATGGGTGTCTCCACA-3′	20	108
	Reverse	5′-CCACGAAGGGGAACTTGTC-3′	19	
ACTA2	Forward	5′-CAGGGCTGTTTTCCCATCCA-3′	20	108
	Reverse	5′-CCTCTTTTGCTCTGTGCTTCGT-3′	22	
RPL22	Forward	5′-TGACATCCGAGGTGCCTTTC-3′	20	101
	Reverse	5′-GTTAGCAACTACGCGCAACC-3′	20	
